# Does the brain listen to the gut?

**DOI:** 10.7554/eLife.17052

**Published:** 2016-05-25

**Authors:** Thomas Kuntz, Jack Gilbert

**Affiliations:** 1 Department of Chemistry, University of Chicago, Chicago, United States; 2 Department of Surgery, University of Chicago, Chicago, United Statesgilbertjack@uchicago.edu

**Keywords:** microbiome, brain, myelin, gut, psychiatry, behavior, Mouse

## Abstract

Transplanting gut bacteria from one mouse strain to another can override genetics and change behavior.

**Related research article** Gacias M, Gaspari S, Mae-Santos P, Tamburini S, Andrade M, Zang F, Shen N, Tolstikov V, Kiebish MA, Dupree JL, Zachariou V, Clemente JC, Casaccia P. 2016. Microbiota-driven transcriptional changes in prefrontal cortex override genetic differences in social behavior. *eLife* **5**:e13442. doi: 10.7554/eLife.13442

**Image** Changes to bacteria in the gut can affect cells in the brain

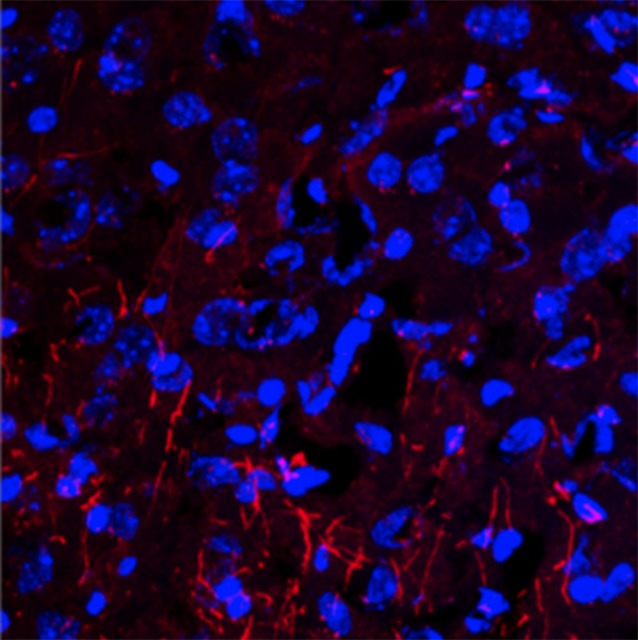

The scientific community has known for centuries that humans are teeming with bacteria. However the emergence of the “microbiome” as a scientific concept represented a turning point in our appreciation of the bacterial world (Lederberg, 2000). The community of microbes that live in our guts (i.e. the gut microbiome) has received particular attention because these microorganisms can dramatically affect our health, both positively and negatively (Turnbaugh et al., 2006). However, it is becoming clearer that the influence of the gut microbiome reaches further than anyone first expected (Foster and McVey Neufeld, 2013).

Now, in eLife, Mar Gacias, Patrizia Casaccia and colleagues report further evidence that microbes in the gut can influence the brain and behavior in mice (Gacias et al., 2016). First, Gacias et al. – who are based at Icahn School of Medicine at Mount Sinai, Virginia Commonwealth University and BERG (a biopharma company) – worked with a mouse strain called NOD (short for non-obese diabetic). Every day for two weeks, one quarter of the mice were fed water through a tube, a quarter were tube-fed a cocktail of antibiotics, a quarter were injected with saline and a quarter were injected with the cocktail of antibiotics (Figure 1). The antibiotics were selected to kill off most of the gut bacteria when given orally but not when injected.

**Figure 1.**
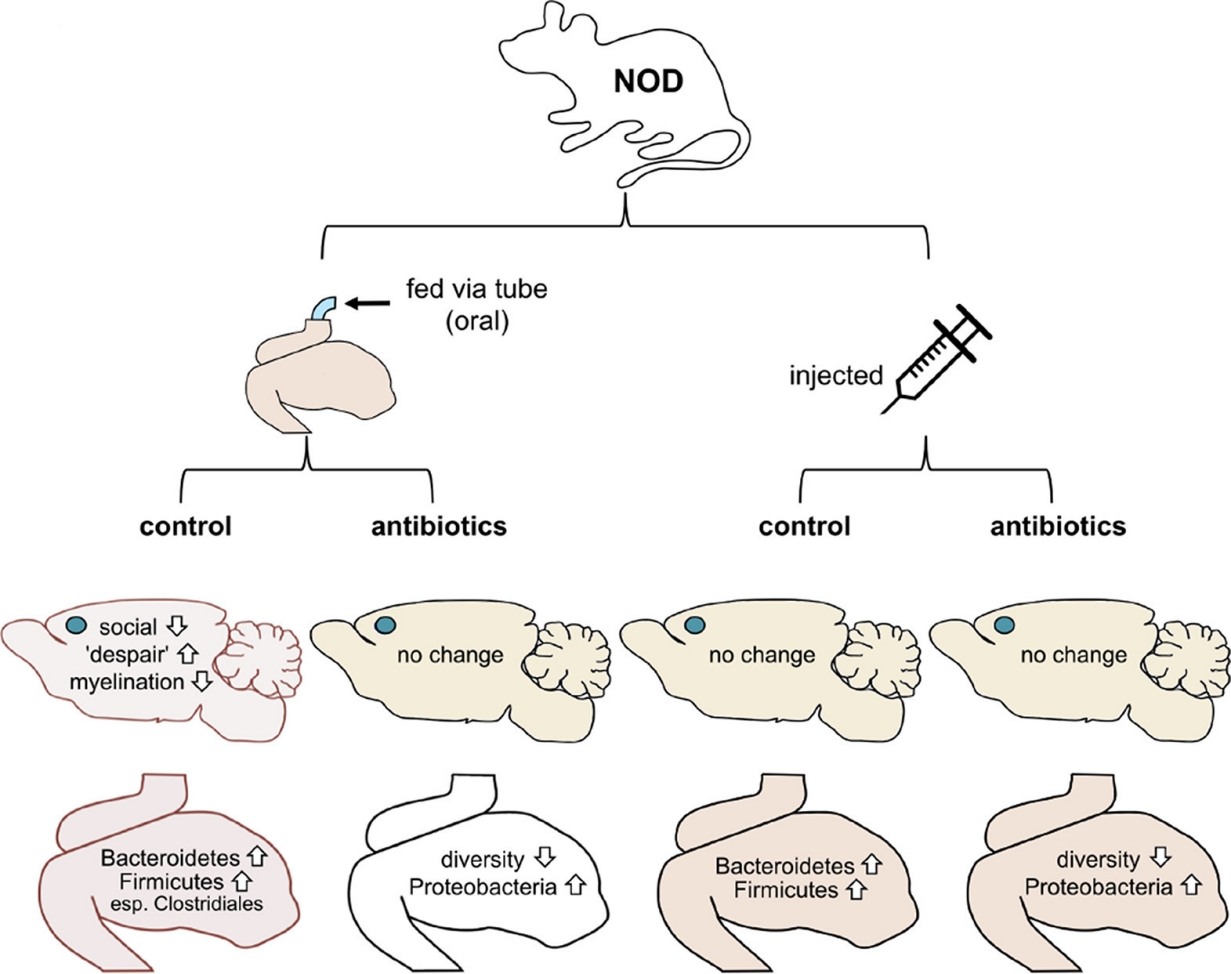
Mouse-based experiments provide further support for the idea that the gut microbiome can influence the brain and behavior. Gacias et al. divided NOD mice into four groups, with each group receiving a different treatment (top). Only mice that received the oral control showed a change in behavior. These mice were less social and showed more "despair-like behaviors". These mice also had reduced myelination in the medial prefrontal cortex (this region’s location in the brain is shown with a blue dot). The gut microbiomes in these mice also became enriched with bacteria called Bacteroidetes and Firmicutes. Oral antibiotics didn’t affect behavior, but did reduce the diversity of the gut microbiome. Injections (control or antibiotics, right) had no effects.

Gacias et al. then tested the behavior of all four groups of NOD mice. Three of the groups did not change their behavior, but strikingly the group that had been fed water through a tube did behave differently. These mice tended to avoid other mice and showed increased signs of "despair-like behaviors" (as assessed using standard tests). Importantly, these behavioral changes did not happen in mice that had been tube-fed antibiotics or in the mice that had been injected instead. Gacias et al. then repeated their tests with a second strain of lab mouse called “B6” (also known as C57BL/6), but none of the treatments affected their behavior.

Since only the tube-fed NOD mice showed differences in behavior, Gacias et al. analyzed them further. They found that the gut microbiome of the control mice (i.e. the mice that had been fed with water) changed slightly, whereas the gut microbiome of the mice that had been fed antibiotics changed more (Figure 1). They also showed that the NOD mice and B6 mice had distinct microbiomes before any treatment.

Gacias et al. then inspected a brain region called the medial prefrontal cortex, which had been linked previously to depression in mouse models of the disorder (Liu et al., 2012). They analyzed how genes were expressed in this region and again found striking differences in the control mice. Genes involved in producing the myelin sheath – which coats nerve fibers and is vital for their activity – were less active in these mice. The control mice also had fewer myelin sheaths in the medial prefrontal cortex than their antibiotic-fed counterparts. Notably, mice that are kept isolated for prolonged periods also produce less myelin in this brain region, and avoid social contact too (Liu et al., 2012). Other studies of germ-free mice have reported that the microbiome can influence myelination as well (Hoban et al., 2016).

Next, Gacias et al. asked if transplanting gut microbes from one mouse to another could trigger the same changes. The answer to this question was yes: B6 mice that received the gut microbes of a NOD mouse donor from the control group became less social and showed more "despair-like behaviors" (Figure 2). This suggests that the microbiome was responsible for the behavioral changes observed.

**Figure 2.**
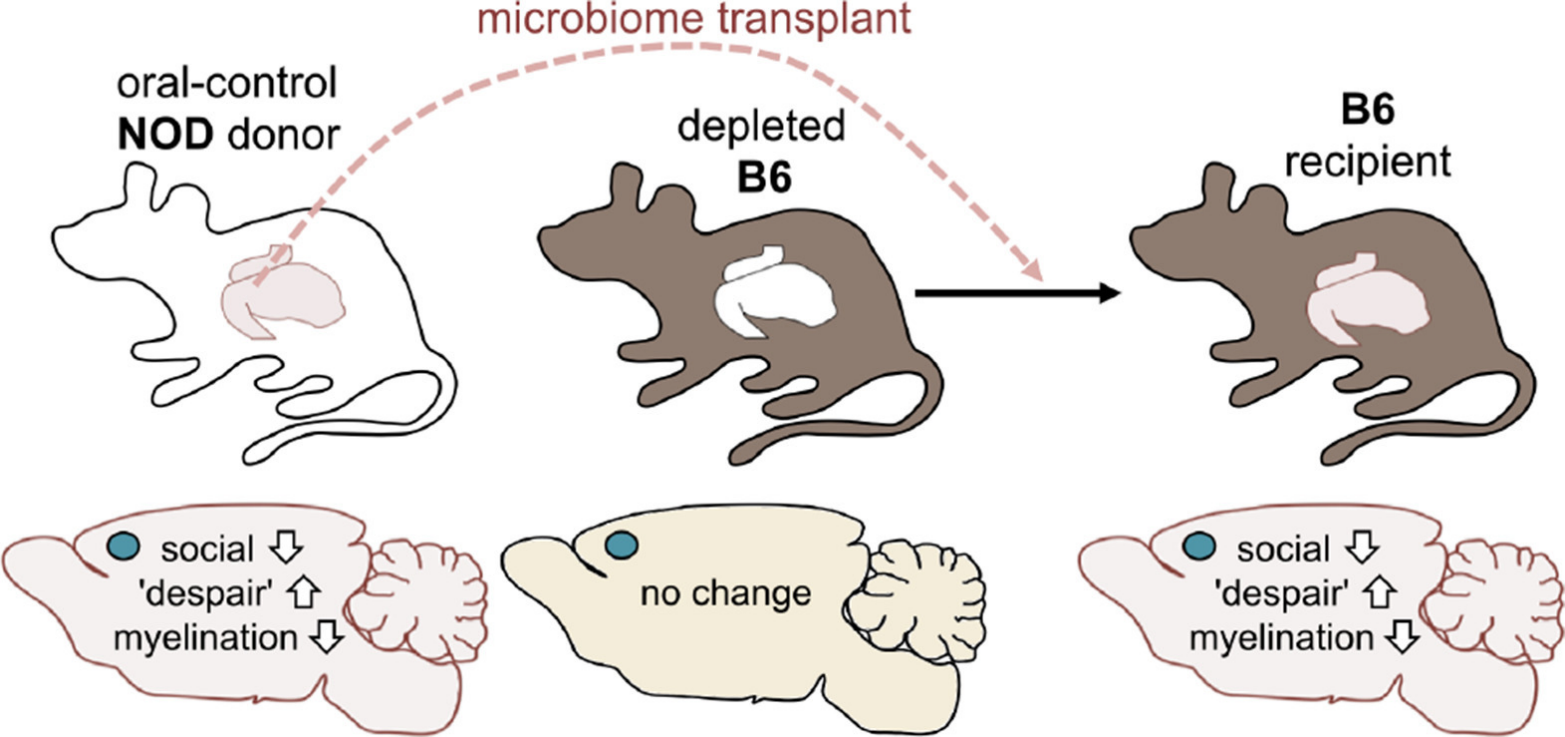
Altering the gut microbiome can trigger behavioral changes. Gacias et al. transplanted the gut microbiome (via fecal transplants) from oral-control NOD mice into B6 mice. The B6 mice had first had their own gut microbiome depleted with antibiotics, and the recipient mice showed similar changes in behavior as the donors. As before, the blue dot indicates the location of the medial prefrontal cortex in the brain.

Gacias et al. also showed that metabolic products found in the gut of B6 mice changed depending on the donor mouse, and that these changes were linked to their behavioral changes too. It is currently not possible to say if these metabolic shifts drove the changes in the medial prefrontal cortex. However, it is known that toxic metabolites from the gut can affect the activity of the central nervous system (Shaw, 2010).

While you should not necessarily be worried about microbial mind-control (Munroe, 2015), these new findings do show that the microbiome can overrule genetics and change both the brain’s chemistry and its activity. The exact mechanism behind this effect still needs to be pinned down. Given the shortage of effective treatments for mental health disorders, exploring how mood-related behavior might be improved via non-traditional means is certainly worthy of further investigation.
